# Anthony Carl (Tony) Kaeser FRCP, FRCPsych: Consultant in Old Age Psychiatry, Basildon sector, Thameside Community Healthcare NHS Trust

**DOI:** 10.1192/pb.bp.116.055079

**Published:** 2017-02

**Authors:** Mike Lowe

**Figure:**
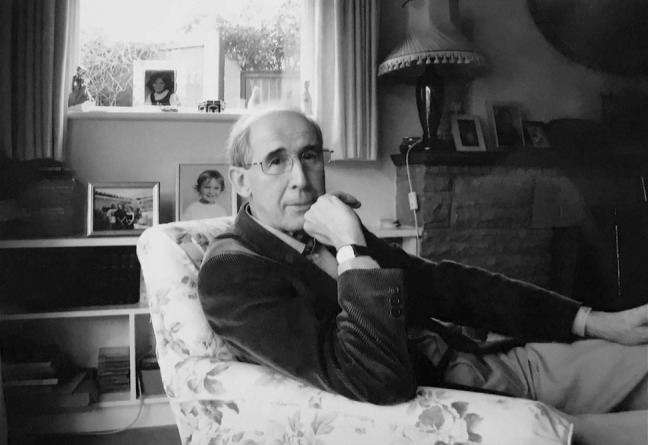


Tony Kaeser, who recently died aged 84, was one of the pioneer consultant psychiatrists who from the 1960s onwards developed departments of psychological medicine in district general hospitals at the time when long-stay mental asylums were being phased out. In the years following his appointment in 1969 as a general adult psychiatrist to Runwell Hospital, he was involved in innumerable planning meetings with the then North East Thames Regional Health Authority to advance the new purpose-built department of psychological medicine at Basildon District General Hospital. This was eventually opened in 1977. It was Tony's attention to detail which improved the ward day areas for the patients – initially they were going to look out on to industrial sites, but with some reconfiguring of the layout the views were transformed to field and countryside vistas.

In the 1980's Tony was appointed convenor for training approval visits for the East Anglian Division of the Royal College of Psychiatrists. He provided liaison psychiatry services at Basildon District General Hospital and was the consultant psychiatrist for the Regional Plastic Surgery and Burns Unit at St. Andrew's Hospital, Billericay for 11 years. He was Chairman of the Basildon General Hospital District Consultant Staff Committee. For the last 7 years of his career he chose to work full time in psychogeriatrics. Despite his heavy clinical load, his professional services were extended willingly to staff and their families from all areas of the National Health Service (NHS) in South Essex.

He was a Foundation member of the Royal College of Psychiatrists. The College recognised his abilities by inviting him in 1984 to act as convenor leading the first team to visit the large Hong Kong training scheme. Shortly before retiring, Tony became a General Medical Council Examiner for doctors with health problems and for a number of years in retirement was one of two Lord Chancellor's Visitors for England. He was also an Area Visitor for the Royal Medical Benevolent Fund until he reached their retirement age.

Tony was born in London in 1932. He qualified at St. Mary's Hospital in 1957 and after house jobs in general medicine he obtained the MRCP and entered the Maudsley training scheme. After obtaining the DPM he was appointed senior registrar at the Maudsley and Institute of Psychiatry. From there he was appointed consultant psychiatrist to Runwell Hospital.

A gentle, kindly, impressively ethical doctor, he was considered in his thinking and conversation but precise, soundly analytical and unfailingly wise. His manners to everyone he encountered were impeccable and right to the end he retained his genuine interest in people. When he became ill himself, he wanted to know about the lives of his carers.

His personal life was varied and fulfilling. He inherited his father's stamp collection. He loved a wide range of music and enjoyed playing his pianola. He joined in regular contract Bridge sessions with medical colleagues throughout his career and retirement, and these only came to an end 4 years before his death. Tony had a great sense of fun and participated in the staff Christmas show for patients. His performances in playing the femme fatale were such a success that he resigned himself to being typecast. He took delight in the achievements of the members of his family. His wife Wendy was an NHS health visitor and they had 2 children and 6 grandchildren, one of whom is a dancer with the Royal Ballet.

In the latter years of his retirement he experienced gradually declining health. He had been diagnosed with a rare hereditary form of amyloid heart disease and although his cognitive faculties were largely undiminished, progressive cardiac failure slowly developed and he died on 18 May 2016. In line with his commitment to humanistic principles, Tony donated his body tissues for the benefit of others and his brain for research into amyloid disease.

